# Supplementary material for: Exploring the mono-/bistability range of positively autoregulated signaling systems in the presence of competing transcription factor binding sites
Source: PLoS Comput Biol. 2022 Nov 22;18(11):e1010738. doi: 10.1371/journal.pcbi.1010738 (PMC9725139; doi:10.1371/journal.pcbi.1010738)
Supplement: S1 Text — (DOCX) [file pcbi.1010738.s001.docx]

**S1 Text. Supplementary methods**

**Model of the autoregulation module**

The autoregulation module describes the autoregulated production of TF protein molecules. For simplicity, transcription and translation are lumped together for TF production with the following two reactions reflecting the basal and induced expression:

$$\begin{aligned} \text{D}_{\text{AR}} \underset{\to}{\alpha} \text{D}_{\text{AR}}+R_{T}+\frac{1}{r} \text{H}\text{K}_{\text{T}} \#\left( R1 \right) \end{aligned}$$

$$\begin{aligned} \text{D}_{\text{AR}}\cdot\text{(RR}_{\text{P}}\text{)}_{\text{2}}\underset{\to}{f\alpha} \text{D}_{\text{AR}}\text{∙}\text{(RR}_{\text{P}}\text{)}_{\text{2}}+R_{T}+\frac{1}{r} \text{H}\text{K}_{\text{T}} .\#\left( R2 \right) \end{aligned}$$

Expression level of histidine kinase HK is treated as a constant proportion of total TF or RR level. The ratio of RR to HK levels is represented by the parameter *r*. Reaction (*R1*) denotes the basal expression of TF and (*R2*) reflects the autoregulated expression with the fold change *f*. D_AR_ and D_AR_ ·(RR_P_)_2_ represent the free and bound forms of the autoregulated promoter. Considering the chromosomal copy of the autoregulated promoter is 1,

$$\begin{aligned} \text{[D}_{\text{AR}}\text{∙}\text{(RR}_{\text{P}}\text{)}_{\text{2}}] + D_{\text{AR}}=1,\#\left( S1 \right) \end{aligned}$$

which is much less than the number of free phosphorylated TF *R_f_*, the binding probability can be derived:

$$\begin{aligned} \text{[D}_{\text{AR}}\text{∙}\text{(RR}_{\text{P}}\text{)}_{\text{2}}\text{]}=\frac{R_{f}^{h}}{R_{f}^{h}+K_{\text{auto}}^{h}}, D_{AR}=\frac{K_{\text{auto}}^{h}}{R_{f}^{h}+K_{\text{auto}}^{h}} .\#\left( S2 \right) \end{aligned}$$

in which *h* is the binding cooperativity and *K*_auto_ is the dissociation constant. The production rate of RR is:

$$\begin{aligned} \text{Production rate}=\alpha* D_{\text{AR}}+f\alpha\text{[D}_{\text{AR}}\text{∙}\text{(RR}_{\text{P}}\text{)}_{\text{2}}]=\alpha\frac{1+f{(R_{f}}/{{K_{\text{auto}})}^{h}}}{1+{(R_{f}}/{{K_{\text{auto}})}^{h}}} .\#\left( S3 \right) \end{aligned}$$

Degradation and dilution of all RR molecules or RR-containing complexes are assumed to occur with the same rate constant *k*_dil_, so the degradation/dilution of all RR-containing molecules (*R_T_*) can be lumped together with the rate –*k*_dil_ *R_T_*, thus the rate change of total RR level is:

$$\begin{aligned} \frac{dR_{T}}{dt}=\alpha\frac{1+f{(R_{f}}/{{K_{\text{auto}})}^{h}}}{1+{(R_{f}}/{{K_{\text{auto}})}^{h}}}-k_{\text{dil}}R_{T} .\#(S4) \end{aligned}$$

Let $R_{b}=\alpha/{k_{\text{dil}}}$ ,

$$\begin{aligned} \frac{dR_{T}}{dt}\cdot\frac{1}{k_{\text{dil}}}=R_{b}\frac{1+f{(R_{f}}/{{K_{\text{auto}})}^{h}}}{1+{(R_{f}}/{{K_{\text{auto}})}^{h}}}-R_{T}=F\left( R_{f} \right)-R_{T}. \end{aligned}$$

At steady state, the rate is zero, thus

$$\begin{aligned} {R_{T}=R}_{b}\frac{1+f{(R_{f}}/{{K_{\text{auto}})}^{h}}}{1+{(R_{f}}/{{K_{\text{auto}})}^{h}}} .\# \end{aligned}$$

When there is no signal or activation, *R_f_* = 0, the total RR level is *R_b_* and the fully induced or maximal *R_T_* value is *fR_b_*. To reduce the free parameters, every concentration parameter is normalized to *K*_auto_, giving:

$$\begin{aligned} R_{T}^{*}=R_{b}^{*}\frac{1+fR_{f}^{*h}}{1+R_{f}^{*h}} .\#\left( S5 \right) \end{aligned}$$

For autoregulated systems with coupled negative feedback, we considered a simple scenario with the phosphorylated TF RR binding to a second binding site within the positively autoregulated promoter and functioning as a repressor of its own transcription. Assuming a similar binding cooperativity *h* as the positive autoregulation, the rate equation is as follows:

$$\begin{aligned} \frac{dR_{T}}{dt}\cdot\frac{1}{k_{\text{dil}}}=R_{b}\left( \frac{1+f{(R_{f}}/{{K_{\text{auto}})}^{h}}}{1+{(R_{f}}/{{K_{\text{auto}})}^{h}}} \right)\left( \frac{K_{N}^{h}+f_{N}R_{f}^{h}}{K_{N}^{h}+R_{f}^{h}} \right)-R_{T}=F\left( R_{f} \right)N\left( R_{f} \right)-R_{T}, \end{aligned}$$

in which *K_N_* is the dissociation constant, *f_N_* is the fold change and the repression function *N*(*R_f_*) corresponds to the term in the second parenthesis. Logarithmic gain of the coupled feedback system, LG*_C_*, can be written as:

$$\begin{aligned} \text{L}\text{G}_{C}=\frac{R_{f}}{F\cdot N}\frac{d (F\cdot N)}{dR_{f}}=\frac{R_{f}}{F}\frac{d F}{dR_{f}}+\frac{R_{f}}{N}\frac{d N}{dR_{f}}=\text{L}\text{G}_{F}+\text{L}\text{G}_{N} . \end{aligned}$$

Normalize all concentrations to *K_auto_*, LG*_N_* can be derived:

$$\begin{aligned} \text{L}\text{G}_{N}=\frac{h\left( f_{N}-1 \right)(\frac{R_{f}^{*}}{K_{N}^{*}})^{h}}{\left( 1+f_{N}(\frac{R_{f}^{*}}{K_{N}^{*}})^{h} \right)\left( 1+(\frac{R_{f}^{*}}{K_{N}^{*}})^{h} \right)} , \text{in which} K_{N}^{*}=\frac{K_{N}}{K_{\text{auto}}} . \#\left( S6 \right) \end{aligned}$$

For auto-repression, the fold change *f_N_* is smaller than 1, thus LG*_N_* is always negative, indicating that the negative autoregulation always decreases the overall LG. The largest decrease, or the minimal LG*_N_* value can be derived:

$$\begin{aligned} \text{minL}\text{G}_{N}=h\frac{\sqrt{f_{N}}-1}{\sqrt{f_{N}}+1} , when R_{f}^{*}={{K_{N}^{*}f}_{N}}^{-1/2h} . \end{aligned}$$

**Model of the DNA binding module**

For many RRs or TFs, each TFBS contains two half-sites and the TF binds as a dimer. We consider the following reactions for binding of phosphorylated RR to the DNA binding site and degradation of DNA·(RR_P_)_2_ complex:

$$\begin{aligned} \text{DN}\text{A}_{\text{i}}+{2R}_{f}\underset{k_{\text{off}}}{\underset{\leftrightarrow}{k_{\text{on}}}} \text{DN}\text{A}_{\text{i}}\text{∙(R}\text{R}_{\text{P}}\text{)}_{\text{2}}\#\left( R3 \right) \end{aligned}$$

$$\begin{aligned} \text{DN}\text{A}_{\text{i}}\text{∙(R}\text{R}_{\text{P}}\text{)}_{\text{2}}\underset{\to}{k_{\text{dil}}} \text{DN}\text{A}_{\text{i}} .\#\left( R4 \right) \end{aligned}$$

The differential rate equation is given by:

$$\begin{aligned} \frac{d\left[ \text{DN}\text{A}_{\text{i}}\text{∙(R}\text{R}_{\text{P}}\text{)}_{\text{2}} \right]}{dt}=k_{\text{on}}[\text{DN}\text{A}_{\text{i}}]R_{f}^{2}-k_{\text{off}}\left[ \text{DN}\text{A}_{\text{i}}\text{∙(R}\text{R}_{\text{P}}\text{)}_{\text{2}} \right]-k_{\text{dil}}\left[ \text{DN}\text{A}_{\text{i}}\text{∙(R}\text{R}_{\text{P}}\text{)}_{\text{2}} \right] .\#\left( S7 \right) \end{aligned}$$

To obtain steady state values, the right side of equation S7 is set to zero, and [DNA_i_] is substituted with $[\text{DN}\text{A}_{\text{i}}]=D-\left[ \text{DN}\text{A}_{\text{i}}\text{∙(R}\text{R}_{\text{P}}\text{)}_{\text{2}} \right]$ in which $D$ is the total concentration of the DNA binding site, yielding the following:

$$\begin{aligned} \left[ \text{DN}\text{A}_{\text{i}}\text{∙(R}\text{R}_{\text{P}}\text{)}_{\text{2}} \right]=D\frac{k_{\text{on}}R_{f}^{2}}{k_{\text{off}}+k_{\text{dil}}+k_{\text{on}}R_{f}^{2}}=D\frac{R_{f}^{2}}{K_{i}^{2}+R_{f}^{2}} \#\left( S8 \right) \end{aligned}$$

in which $K_{i}^{2}=(k_{\text{off}}+k_{\text{dil}})/k_{\text{on}}$. For stable TF proteins, *k*_dil_ is the growth dilution rate constant, usually much smaller than the off rate *k*_off_ of the fast DNA association/dissociation reaction, then $K_{i}\cong{(k}_{\text{off}}/k_{\text{on}})^{1/2}=K_{d}$. For binding reaction with a different cooperativity *l*, eq. (S8) can be written as:

$$\begin{aligned} \left[ \text{DN}\text{A}_{\text{i}}\text{∙(R}\text{R}_{\text{P}}\text{)}_{\text{2}} \right]= D\frac{R_{f}^{l}}{K_{i}^{l}+R_{f}^{l}} .\#\left( S9 \right) \end{aligned}$$

We define *R_P_* as the sum of free *RR_P_* molecules (*R_f_*) and all the *RR_P_* molecules in bound DNA complexes:

$$\begin{aligned} R_{P}=R_{f}+2D\frac{R_{f}^{l}}{K_{i}^{l}+R_{f}^{l}}+2\cdot\frac{R_{f}^{h}}{R_{f}^{h}+K_{\text{auto}}^{h}} .\#\left( S10 \right) \end{aligned}$$

Because the number of binding sites in the autoregulated promoter is usually much less than the number of free phosphorylated TF molecules, the last term corresponding to binding of the autoregulated promoter is ignored. To compare with the autoregulation module, parameters are normalized by *K*_auto_ as the following:

$$R_{P}^{*}=\frac{R_{P}}{K_{\text{auto}}}, R_{f}^{*}=\frac{R_{f}}{K_{\text{auto}}}, D^{*}=\frac{D}{K_{\text{auto}}}, K^{*}=\frac{K_{i}}{K_{\text{auto}}} .$$

From eq. (S10), we obtain:

$$\begin{aligned} R_{P}^{*}=R_{f}^{*}+2D^{*}\frac{R_{f}^{*l}}{K^{*l}+R_{f}^{*l}} .\#\left( S11 \right) \end{aligned}$$

The logarithmic gain can be calculated from eq. (S11):

$$\text{L}\text{G}_{B}=\frac{R_{P}^{*}}{R_{f}^{*}} \frac{{dR}_{f}^{*}}{dR_{P}^{*}}=\frac{(R_{f}^{*l}+K^{*l})((R_{f}^{*l}+K^{*l}+2D^{*}R_{f}^{*l-1})}{(R_{f}^{*l}+K^{*l})^{2}+2D^{*}lK^{*l}R_{f}^{*l-1}}$$

$$\begin{aligned} =1+\frac{2D^{*}R_{f}^{*l-1}\left( R_{f}^{*l}-\left( l-1 \right)K^{*l} \right)}{(R_{f}^{*l}+K^{*l})^{2}+2D^{*}lK^{*l}R_{f}^{*l-1}} .\#\left( S12 \right) \end{aligned}$$

LG*_B_* needs to higher than 1 to promote bistability. For $\text{L}\text{G}_{B}=1$, it requires either $D^{*}=0$ or $R_{f}^{*l}-\left( l-1 \right)K^{*l}=0$, thus

$$\begin{aligned} R_{f}^{*}=(l-1)^{\frac{1}{l}}K^{*} .\#\left( S13 \right) \end{aligned}$$

When *l*=2, this becomes $R_{f}^{*}=K^{*}$, which has been described in the main text. Any *R_f_^*^*values higher than that shown in (S13) could potentially lead to higher overall gain. Eq. (S12) is not limited to cooperative binding with each site binding to two RR molecules. For binding with a different stoichiometry *n*, the term *2D** can be changed to *nD** to account for the binding stoichiometry. And non-cooperative binding (*l*=1) can also lead to LG*_B_* larger than 1, promoting bistability.

To evaluate the maximal value of LG*_B_*, eq. (S12) is rewritten as:

$$\begin{aligned} \text{L}\text{G}_{B}=1+\frac{(\frac{2D^{*}}{K^{*}})\gamma\left( 1-\left( l-1 \right)\gamma^{l} \right)}{(1+\gamma^{l})^{2}+(\frac{2D^{*}}{K^{*}})l\gamma^{l+1}} , \text{ in which }\gamma=\frac{K^{*}}{R_{f}^{*}} .\# \end{aligned}$$

Solve for $d\text{L}\text{G}_{B}/d\gamma=0$, we can derive:

$$(l-1)^{2}\gamma^{3l}-\left( 4l-3 \right)\gamma^{2l}-\frac{2D^{*}}{K^{*}}l^{2}\gamma^{l+1}-\left( l^{2}+2l-3 \right)\gamma^{l}+1=0.$$

Solutions for the above equation can be numerically obtained for specific *D**, *K** and *l* values. When *l* = 2, for values of *D** and *K** shown in Fig 3C (*D** =1, *K** =0.33, 0.5, 1, 3), the maximal LG*_B_* occurs at *γ* = 0.29, 0.31, 0.35, 0.39. Based on the definition of *γ*, occupancy of the TFBSs equals $1/{(1+\gamma^{l})}$. Thus, when LG*_B_* reaches the maximum, the corresponding TFBS occupancy is 0.92, 0.91, 0.89 and 0.87, respectively. It appears that the logarithmic gain reaches the maximum when most TFBSs are bound.

**Model of the phosphorylation module**

The phosphorylation module is based on the model described previously [1, 2]. As shown in Fig 4A, the following reactions are considered:

$$\begin{aligned} \text{HK} \underset{k_{\text{-k}}}{\underset{\rightleftharpoons}{k_{k}}} \text{H}\text{K}_{\text{P}} \#\left( R5 \right) \end{aligned}$$

$$\begin{aligned} \text{H}\text{K}_{\text{P}}+\text{RR} \underset{k_{\text{-1}}}{\underset{\rightleftharpoons}{k_{1}}} \text{H}\text{K}_{\text{P}}\text{∙RR}\underset{\to}{k_{t}} \text{HK}+\text{R}\text{R}_{\text{P}}\#\left( R6 \right) \end{aligned}$$

$$\begin{aligned} \text{HK}+\text{R}\text{R}_{\text{P}} \underset{k_{\text{-2}}}{\underset{\rightleftharpoons}{k_{2}}} \text{HK∙R}\text{R}_{\text{P}}\underset{\to}{k_{p}} \text{HK}+\text{RR} .\#\left( R7 \right) \end{aligned}$$

Assuming individual species having the same degradation/growth-dilution rate constant, the differential equations are:

$$\begin{aligned} \frac{d \left[ \text{H}\text{K}_{\text{P}}\text{∙RR} \right]}{dt}=k_{1}\left[ \text{H}\text{K}_{\text{P}} \right]\left[ \text{RR} \right]-\left( k_{\text{-1}}+k_{t}+k_{\text{dil}} \right)\left[ \text{H}\text{K}_{\text{P}}\text{∙RR} \right] \#\left( S14 \right) \end{aligned}$$

$$\begin{aligned} \frac{d \left[ \text{HK∙R}\text{R}_{\text{P}} \right]}{dt}=k_{2}\left[ \text{HK} \right]\left[ \text{R}\text{R}_{\text{P}} \right]-\left( k_{\text{-2}}+k_{p}+k_{\text{dil}} \right)\left[ \text{HK∙R}\text{R}_{\text{P}} \right] \#\left( S15 \right) \end{aligned}$$

$$\begin{aligned} \frac{d \left[ \text{H}\text{K}_{\text{P}} \right]}{dt}=k_{k}\left[ \text{HK} \right]-(k_{\text{-k}}+k_{\text{dil}})\left[ \text{H}\text{K}_{\text{P}} \right]- k_{1}\left[ \text{H}\text{K}_{\text{P}} \right]\left[ \text{RR} \right]+k_{\text{-1}}\left[ \text{H}\text{K}_{\text{P}}\text{∙RR} \right] \#\left( S16 \right) \end{aligned}$$

$$\begin{aligned} \frac{d \left[ \text{R}\text{R}_{\text{P}} \right]}{dt}={-k}_{2}\left[ \text{HK} \right]\left[ \text{R}\text{R}_{\text{P}} \right]+k_{\text{-2}}\left[ \text{HK∙R}\text{R}_{\text{P}} \right]+k_{t}\left[ \text{H}\text{K}_{\text{P}}\text{∙RR} \right]-k_{\text{dil}}\left[ \text{R}\text{R}_{\text{P}} \right]\#\left( S17 \right) \end{aligned}$$

$$\begin{aligned} \frac{d \left[ \text{RR} \right]}{dt}={-k}_{1}\left[ \text{H}\text{K}_{\text{P}} \right]\left[ \text{RR} \right]+k_{\text{-1}}\left[ \text{H}\text{K}_{\text{P}}\text{∙RR} \right]+k_{p}\left[ \text{HK∙R}\text{R}_{\text{P}} \right]-k_{\text{dil}}\left[ \text{RR} \right] .\#\left( S18 \right) \end{aligned}$$

At steady state, all the rates equal to zero. Eq. (S14) and (S15) can give:

$$\begin{aligned} \left[ \text{H}\text{K}_{\text{P}}\text{∙RR} \right]=\frac{k_{1}}{k_{\text{-1}}+k_{t}+k_{\text{dil}}}\left[ \text{H}\text{K}_{\text{P}} \right]\left[ \text{RR} \right]=\frac{1}{K_{\text{Mt}}}\left[ \text{H}\text{K}_{\text{P}} \right]\left[ \text{RR} \right] \#\left( S19 \right) \end{aligned}$$

$$\begin{aligned} \left[ \text{HK∙R}\text{R}_{\text{P}} \right]=\frac{k_{2}}{k_{\text{-2}}+k_{p}+k_{\text{dil}}}\left[ \text{HK} \right]\left[ \text{R}\text{R}_{\text{P}} \right]=\frac{1}{K_{\text{Mp}}}\left[ \text{HK} \right]\left[ \text{R}\text{R}_{\text{P}} \right]\#\left( S20 \right) \end{aligned}$$

where *K*_Mp_ and *K*_Mt_ are defined as:

$$K_{\text{Mt}}=\frac{k_{\text{-1}}+k_{t}+k_{\text{dil}}}{k_{1}} , K_{\text{Mp}}=\frac{k_{\text{-2}}+k_{p}+k_{\text{dil}}}{k_{2}} .$$

Substituting eq. (S19) into (S16), we can derive:

$$\begin{aligned} \left[ \text{H}\text{K}_{\text{P}} \right]=\frac{k_{k}\left[ \text{HK} \right]}{\left( k_{\text{-k}}+k_{\text{dil}} \right)+\frac{k_{t}+k_{\text{dil}}}{K_{\text{Mt}}} \left[ \text{RR} \right]}=\frac{K_{\text{Mt}}}{k_{t}+k_{\text{dil}}} \frac{k_{k}\left[ \text{HK} \right]}{Ct+\left[ \text{RR} \right]} \#\left( S21 \right) \end{aligned}$$

where *Ct* is defined as:

$$\begin{aligned} Ct=K_{Mt}\frac{k_{\text{-k}}+k_{\text{dil}}}{k_{t}+k_{\text{dil}}} .\#\left( S22 \right) \end{aligned}$$

Adding eq. (S14), (S15) and (S17) together and substituting (S19), (S20) and (S21) gives:

$$\begin{aligned} \left[ RR_{P} \right]=(\frac{k_{k}}{Cp}+\frac{k_{\text{dil}}}{\left[ \text{HK} \right]})^{-1}\left( \frac{k_{t}}{k_{t}+k_{\text{dil}}} \right)\frac{k_{k}\left[ \text{RR} \right]}{Ct+\left[ \text{RR} \right]} ,\#\left( S23 \right) \end{aligned}$$

where *Cp* is defined as:

$$\begin{aligned} Cp=K_{\text{Mp}}\frac{k_{k}}{k_{p}+k_{\text{dil}}} .\#\left( S24 \right) \end{aligned}$$

Eq. (S23) is similar to the result derived earlier [2] except that parameters are re-defined to consider the dilution/degradation rate.

Many TCSs have displayed fast HK-autophosphorylation and phosphotransfer rates with half-times in minutes [3]. When the degradation/growth-dilution rate is slow, with the rate constant *k*_dil_ much smaller than the rate constants of all phosphorylation/dephosphorylation reaction rates, *k*_dil_ can be ignored in all above equations, and definitions of *Cp*, *Ct*, *K*_Mp_, *K*_Mt_ will be the same as the model described previously [1, 2]:

$$K_{\text{Mt}}=\frac{k_{\text{-1}}+k_{t}}{k_{1}} , K_{\text{Mp}}=\frac{k_{\text{-2}}+k_{p}}{k_{2}}, Cp=K_{\text{Mp}}\frac{k_{k}}{k_{p}}, Ct=K_{\text{Mt}}\frac{k_{\text{-k}}}{k_{t}} .$$

Eq. (S23) will become eq. (7) in the main text when $Cp\ll k_{k}[\text{HK}]/k_{\text{dil}}$:

$$\begin{aligned} \left[ \text{R}\text{R}_{\text{P}} \right]=Cp\frac{\left[ \text{RR} \right]}{Ct+\left[ \text{RR} \right]} . \end{aligned}$$

When the total RR concentration is much greater than the total concentration of HK, concentrations of complexes are ignored, $\left[ \text{RR} \right]\cong R_{T}-\left[ \text{R}\text{R}_{\text{P}} \right]$, giving

$$\begin{aligned} \left[ \text{R}\text{R}_{\text{P}} \right]\cong Cp\frac{R_{T}-\left[ \text{R}\text{R}_{\text{P}} \right]}{Ct+R_{T}-\left[ \text{R}\text{R}_{\text{P}} \right]} .\#(S25) \end{aligned}$$

The modeling above is based on the phosphorylation module only, assuming the phosphorylation module is decoupled from the DNA binding module. Thus, [RR_P_] corresponds to *R_P_* in the main text. Binding of RR_P_ to TFBSs can potentially impact the phosphorylation module by sequestering RR_P_ from binding to the HK for the dephosphorylation reaction. However, the RR uses different surfaces from different domains for interactions with the HK and DNA. It is not known whether binding to DNA can interfere with the HK interaction. For simplicity, here we assume that the HK has the same binding affinity and phosphatase activity toward DNA-bound RR_P_ as free RR_P_ (*R_f_*), minimizing the phosphatase sequestration effect by DNA. Thus, [RR_P_] in eq. (S25) corresponds to the sum of free and DNA-bound RR_P_, which is the input *R_P_* for the DNA binding module.

The approximation shown in eq. (S25) is the basis for modeling the phosphorylation module described in the main text. When TCSs have a low ratio of RR/HK and the total RR concentration is not in great excess to HK, eq. (S14-S18) and the mass conservation eq. (14) in the main text are combined to numerically solve for the steady states using the simbiology tool of Matlab 2019a. Parameter values used for the full model are as follows: *k_-1_* and *k_-2_*, 0.6 s^-1^; *k_1_* and *k_2_*, 0.001 molecule^-1^s^-1^; *k_k_* and *k_-k_*, 0.03 s^-1^; *k_p_*, 0.01 s^-1^; *k_t_*, 0.1 s^-1^.

To derive the logarithmic gain of the phosphorylation module, eq. (16) in main text was differentiated in respect to *R_T_*,

$$\begin{aligned} \text{L}\text{G}_{A}=\frac{R_{T}^{*}}{R_{P}^{*}}\frac{dR_{P}^{*}}{dR_{T}^{*}}=\frac{R_{T}^{*}}{2R_{P}^{*}}\left( 1-\frac{Ct^{*}-Cp^{*}+R_{T}^{*}}{X_{P}^{*}} \right)\#\left( S26 \right) \end{aligned}$$

in which $X_{P}^{*}=X_{P}/K_{auto}$ and *X_P_* is defined as:

$$\begin{aligned} X_{P}=\sqrt{(Cp+Ct+R_{T})^{2}-4CpR_{T}}=Cp+Ct+R_{T}-2R_{P} .\#\left( S27 \right) \end{aligned}$$

From eq. (15), we can derive:

$$\begin{aligned} \frac{R_{T}^{*}}{R_{P}^{*}}=\frac{Cp^{*}+Ct^{*}-R_{P}^{*}}{Cp^{*}-R_{P}^{*}} .\#\left( S28 \right) \end{aligned}$$

Substituting Eq. (S27) and (S28) into (S26) gives:

$$\begin{aligned} \text{L}\text{G}_{A}=\frac{Cp^{*}+Ct^{*}-R_{P}^{*}}{Cp^{*}+Ct^{*}+R_{T}^{*}-2R_{P}^{*}}=1-\frac{R_{T}^{*}-R_{P}^{*}}{\left( Cp^{*}+Ct^{*}-R_{P}^{*} \right)+R_{T}^{*}-R_{P}^{*}} . \#\left( S29 \right) \end{aligned}$$

Eq. (S29) is used to analyze how the composite parameter *Cp* and *Ct* affect the overall logarithmic gain and the bistability range. From eq. (S28), we can obtain the fraction of phosphorylated RR:

$$\begin{aligned} \frac{R_{P}^{*}}{R_{T}^{*}}=\frac{Cp^{*}-R_{P}^{*}}{Cp^{*}+Ct^{*}-R_{P}^{*}} .\#\left( S30 \right) \end{aligned}$$

When $R_{T}^{*}=2\left( Cp^{*}-Ct^{*} \right),$phosphorylation fraction is 1/2 if $Cp^{*}>Ct^{*}$. If $Cp^{*}<Ct^{*}$, phosphorylation fraction will be always smaller than 1/2.

**Steady-state transcriptional responses**

Eqs. (S5), (S11) and (16) are used to numerically solve the steady-state solutions of *R_f_^*^*, *R_P_^*^* and *R_T_^*^*. Transcriptional responses are modeled as occupancy of the promoter of interest:

$$\begin{aligned} \text{occupancy}=\frac{R_{f}^{*2}}{K_{\text{rep}}^{* 2}+R_{f}^{*2}} ,\#\left( S31 \right) \end{aligned}$$

assuming a binding cooperativity of 2. *K^*^*_rep_ represents the TF binding affinity to the promoter of interest. For the autoregulated promoter, *K^*^*_rep_=1. In our model, we consider a single affinity *K^*^* for all other TFBSs for simplicity. Among these TFBSs are the binding sites for TF-regulated promoters. For these TF target promoters, *K^*^*_rep_= *K^*^*. Promoter occupancy depends on both *K^*^*_rep_ and *R_f_^*^*. Higher *K^*^*_­_ values lead to less TFBS competition and higher *R_f_^*^*, while higher values of *K^*^*_rep_ result in lower occupancy. Thus, the transcriptional response has a complex dependence on multiple parameters (S5 Fig).

Information capacity, or signaling capacity, has been developed to measure the capability of a signaling system to resolve different levels of input [4]. The signaling capacity is dependent on the fraction of the system being activated at saturating stimulus levels. For a given promoter, promoter occupancy can be used to assess the signaling capacity. It is apparent that increase of binding competition with a lower *K^*^* value will always lower the promoter occupancy (comparing red to purple, or blue to brown lines in S5C and S5D), thus reduce the signaling capacity.

**Cloning of strains and plasmids**

The CusR DNA binding site sequence is based on previous characterization with DNase-I footprinting experiments [5]. The CusR binding site (bold letters) is located in the intergenic region between *cusR* and *cusC*.

CusR Binding Site -35 *PcusC*

*PcusR*...TGGCAATCGCTTATTGGC**AAAATGACAATTTTGTCATTTT**TCTGTCACCGGAAAAT...*PcusC*

*PcusR* -35

The binding site was created by annealing two oligos containing the reported CusR site flanked by non-specific sequences. The annealed oligo has complementary overhangs to allow concatenation into a higher number of repeated sites. After self-ligating the annealed oligos, the resulting DNA was ligated to pRG475 with annealed adapter DNA. The resulting plasmids were screened for different numbers of CusR binding sites, yielding pCusRBS1, pCusRBS2 and pCusRBS3. All decoy plasmids carry an arabinose-inducible *rop* gene that can inhibit plasmid replication.

cusRBSs: 5’-ctgc CACGCCGAAGCGGAGC **AAAATGACAATTTTGTCATTTTT** CCAAGCCCTAGAAGC

cusRBSr: GTGCGGCTTCGCCTCG **TTTTACTGTTAAAACAGTAAAAA** GGTTCGGGATCTTCG gacg-5’

Adapter1s: 5’- gaccATGCTGACCCGGGGAGGG

Adapter1r: TACGACTGGGCCCCTCCC gacg-5’

Adapter2s: 5’- ctgcCCACCGGGAAGGCTTGAGCT

Adapter2r: GGTGGCCCTTCCGAACTCGA cgaa-5’

Binding affinity or cooperativity of the CusR site has not been characterized. The CusR binding site was suggested to contain two half sites (underlined) in a head-to-head orientation [5], which is different from typical OmpR subfamily of RRs. SELEX studies indicate that phosphorylated CusR can bind DNA in a head-to-tail orientation as typical of OmpR subfamily RRs [6]. It appears that an additional half-site (dotted) may exist downstream of the current identified site, which can potentially impact the cooperativity and affinity. Therefore, the engineered decoy site on plasmids contains only a truncated version of the WT sequence and may have a binding affinity and cooperativity different from the WT genomic site.

To create the chromosomal reporter strain, the *cusC* promoter, an engineered ribosome binding site (RBS), mGreenLantern gene, and a plasmid derived from pAH144 [7] were assembled by golden gate cloning. The resulting plasmid pDonorZb-GL contains the *PcusC-mGreenLantern*, and the bacterial attachment site that allows plasmid integration into the HK022 phage attachment site of the chromosome. Plasmid DNA was integrated into the genome and it was confirmed only a single copy of the plasmid was integrated. The sequence corresponding to the promoter, RBS and start codon of mGreenLantern is shown below.

BsaI for cloning

GGAGACCATGTTGCCCGGGCAATTCTAGAGTAGCGGGATCAGATGGCAATCGCTTATTGGC**AAAATGACA**

-35 -10 +1 BsaI

**ATTTTGTCATTTT**TCTGTCACCGGAAAATCAGAGCCTGGCGAGTAAAGTTGGCGGCGGTCTCTAGAACGA

RBS start

AATTCAATAAGGAGGAGGTGTTAA**ATG**GTG

**REFERENCE**

1. Batchelor E, Goulian M. Robustness and the cycle of phosphorylation and dephosphorylation in a two-component regulatory system. Proc Natl Acad Sci USA. 2003;100(2):691-6. doi: 10.1073/pnas.0234782100. PMID: 12522261.

2. Siryaporn A, Perchuk BS, Laub MT, Goulian M. Evolving a robust signal transduction pathway from weak cross-talk. Mol Syst Biol. 2010;6:452. doi: 10.1038/msb.2010.105. PMID: 21179024.

3. Yamamoto K, Hirao K, Oshima T, Aiba H, Utsumi R, Ishihama A. Functional characterization *in vitro* of all two-component signal transduction systems from *Escherichia coli*. J Biol Chem. 2005;280(2):1448-56. doi: 10.1074/jbc.M410104200. PMID: 15522865.

4. Komorowski M, Tawfik DS. The limited information capacity of cross-reactive sensors drives the evolutionary expansion of signaling. Cell Syst. 2019;8(1):76-85 e6. doi: 10.1016/j.cels.2018.12.006. PMID: 30660612.

5. Yamamoto K, Ishihama A. Transcriptional response of *Escherichia coli* to external copper. Mol Microbiol. 2005;56(1):215-27. doi: 10.1111/j.1365-2958.2005.04532.x. PMID: 15773991.

6. Joyce AP, Havranek JJ. Deciphering the protein-DNA code of bacterial winged helix-turn-helix transcription factors. Quantitative Biology. 2018;6(1):68-84. doi: 10.1007/s40484-018-0130-0.

7. Haldimann A, Wanner BL. Conditional-replication, integration, excision, and retrieval plasmid-host systems for gene structure-function studies of bacteria. J Bacteriol. 2001;183(21):6384-93. doi: 10.1128/JB.183.21.6384-6393.2001. PMID: 11591683.
