# Supplementary figures and images for: Exploring the mono-/bistability range of positively autoregulated signaling systems in the presence of competing transcription factor binding sites
Source: PLoS Comput Biol. 2022 Nov 22;18(11):e1010738. doi: 10.1371/journal.pcbi.1010738 (PMC9725139; doi:10.1371/journal.pcbi.1010738)

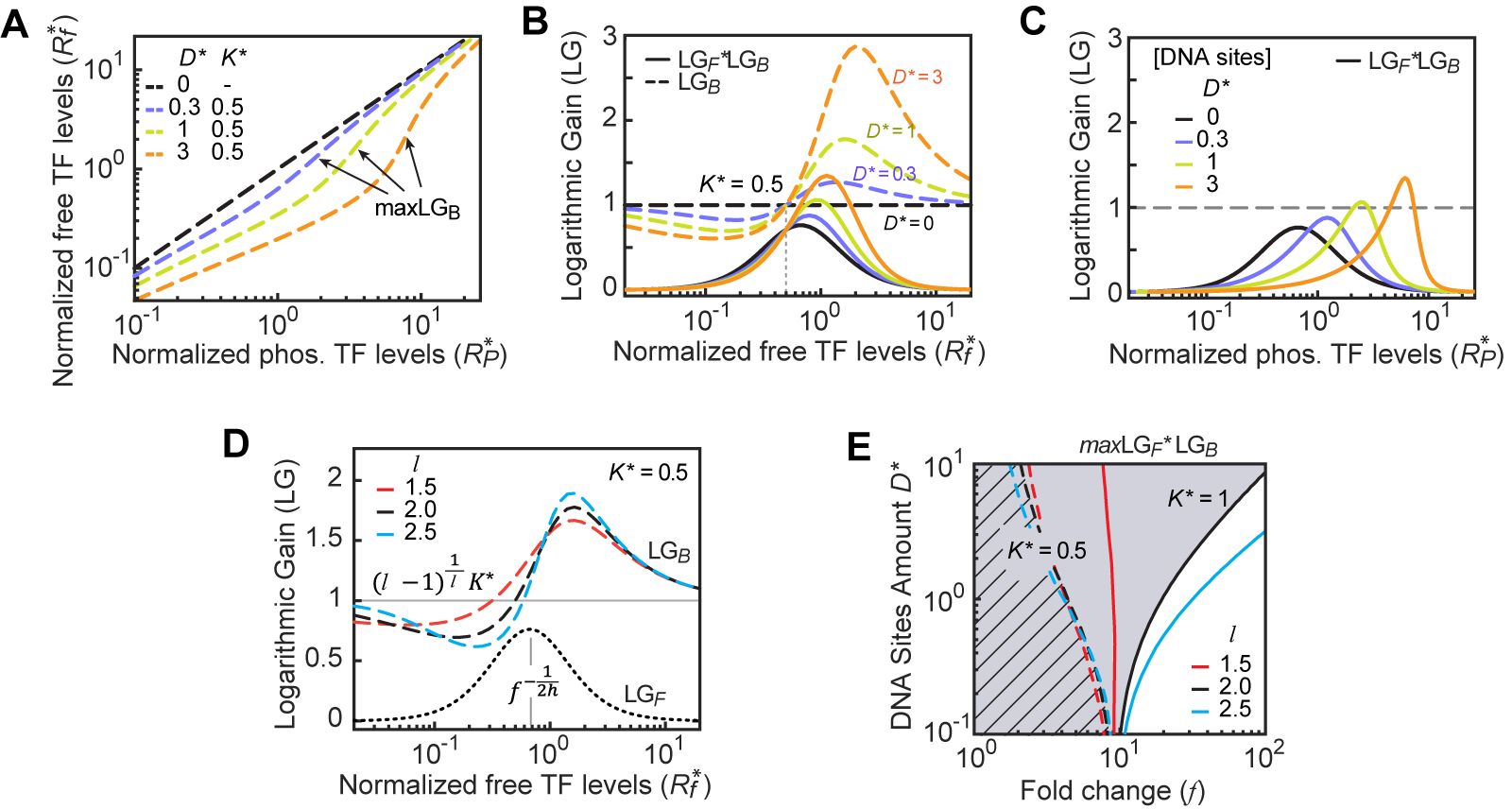

Supplement: S1 Fig — (A) Functional dependence of Rf* on Rp* with different amounts of TFBSs D*. Arrows indicate where the maxima of LGB occur. (B and C) Increase of LGs by increased amounts of competing TFBSs. Dashed lines represent LGs of the binding module. Solid lines represent the combined LGs from the autoregulation and DNA binding modules, which are plotted on the scales of Rf* (B) and Rp* (C). (D) Differences in LGB with different binding cooperativity of competing TFBSs. Lowering TFBS cooperativity l reduces the LGB maximum but expands the region with LGB >1. The formula indicates the Rf* value where LGB = 1. The dotted line shows the LGF from the autoregulation module as reference for aligning with LGB. (E) A phase diagram showing the monostability range with different binding cooperativity l. The gray shaded area shows the monostability range (maxLGFLGB<1) with K* = 1 and l = 2. The striped region shows the corresponding monostability range with K* = 0.5 and l = 2. Colored lines represent the contour lines with maxLGFLGB = 1 at different l values and they indicate the borders of the mono-/bistability ranges. (TIF) [file pcbi.1010738.s002.tif]

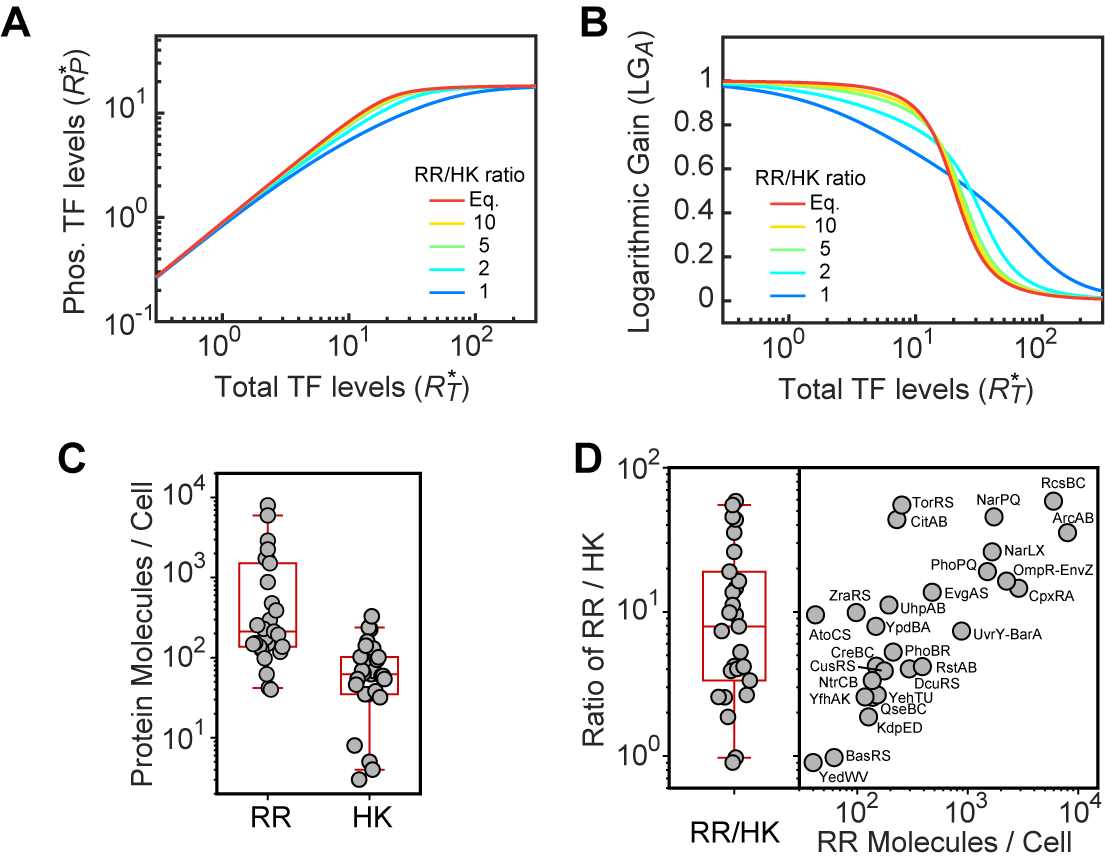

Supplement: S2 Fig — (A and B) Influences of RR/HK ratios on RR phosphorylation (A) and logarithmic gain LGA (B). Red lines illustrate the corresponding Rp* and LGA values obtained from Eqs (16) and (17) with our approximation model discussed in the main text, assuming the RR is in great excess to the HK. Colored lines represent simulated data with the indicated RR/HK ratios using the full model described in S1 Text. Significant deviation from the approximation model is only apparent with an RR/HK ratio of 1. Parameter values are as follows: Cp*, 18.3; Ct*, 2.1; Kauto, 100 molecules/cell (10−15 L). (C and D) Protein molecule numbers of RRs and HKs in E. coli. Protein abundance data were obtained from ribosome profiling of the E. coli proteome [29] to derive the RR/HK ratios (D). Most of the RR/HK pairs have a ratio greater than 1. (TIF) [file pcbi.1010738.s003.tif]

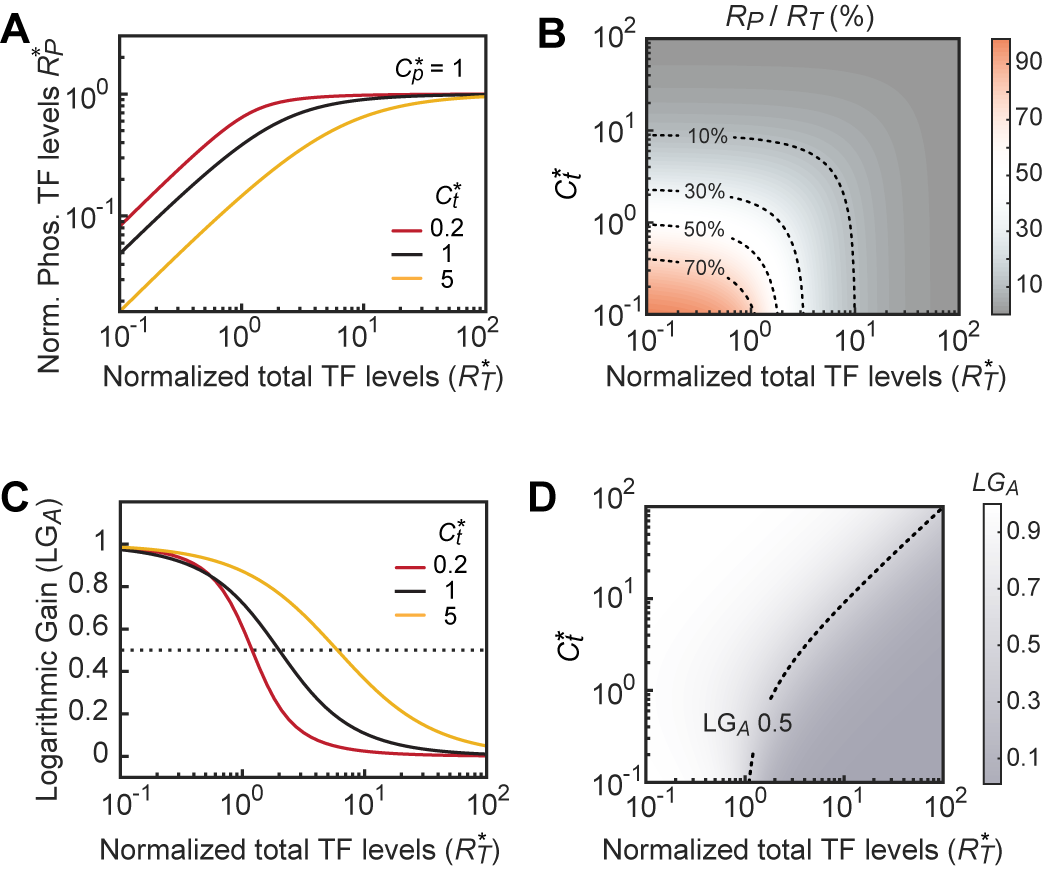

Supplement: S3 Fig — Ct* is negatively correlated with the phosphorylation level Rp* (A) and fractions of phosphorylated RR (B). Lower Ct* values lead to higher phosphorylation levels before saturation. (C and D) Effects of Ct* on the logarithmic gain LGA. Dotted lines indicate the parameter values that give LGA = 0.5. Low LGA values (<0.5) can limit the LG of the autoregulation module regardless of the fold change f. All graphs are generated with Cp* = 1. (TIF) [file pcbi.1010738.s004.tif]

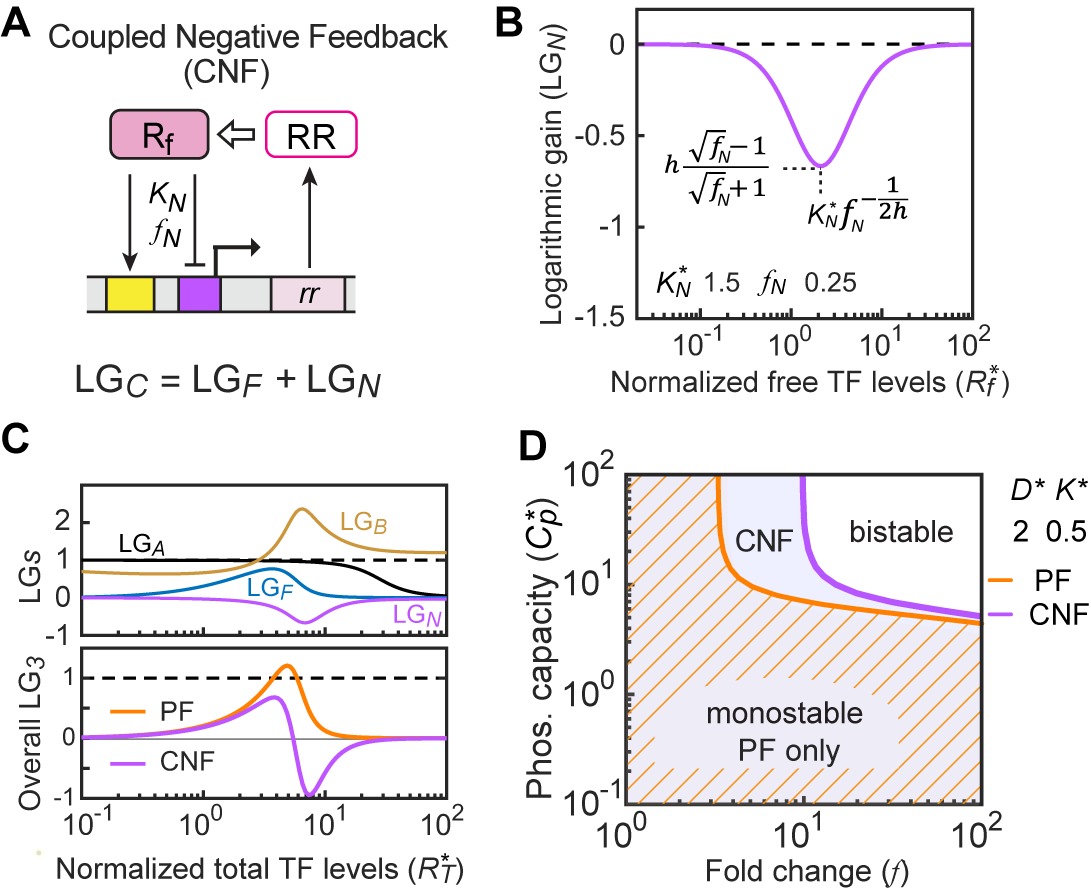

Supplement: S4 Fig — (A) Illustration of a simple negative autoregulation scheme. A phosphorylated RR binds to the repression site within its own promoter with an affinity of KN, leading to transcriptional repression with a fold change fN. (B) The logarithmic gain of the negative autoregulation module. Because LGN is always negative, the coupled negative feedback (CNF) will lower the overall LG3 (C) and shrink the bistability range (D) in comparison to the system with only a positive feedback (PF). (TIF) [file pcbi.1010738.s005.tif]

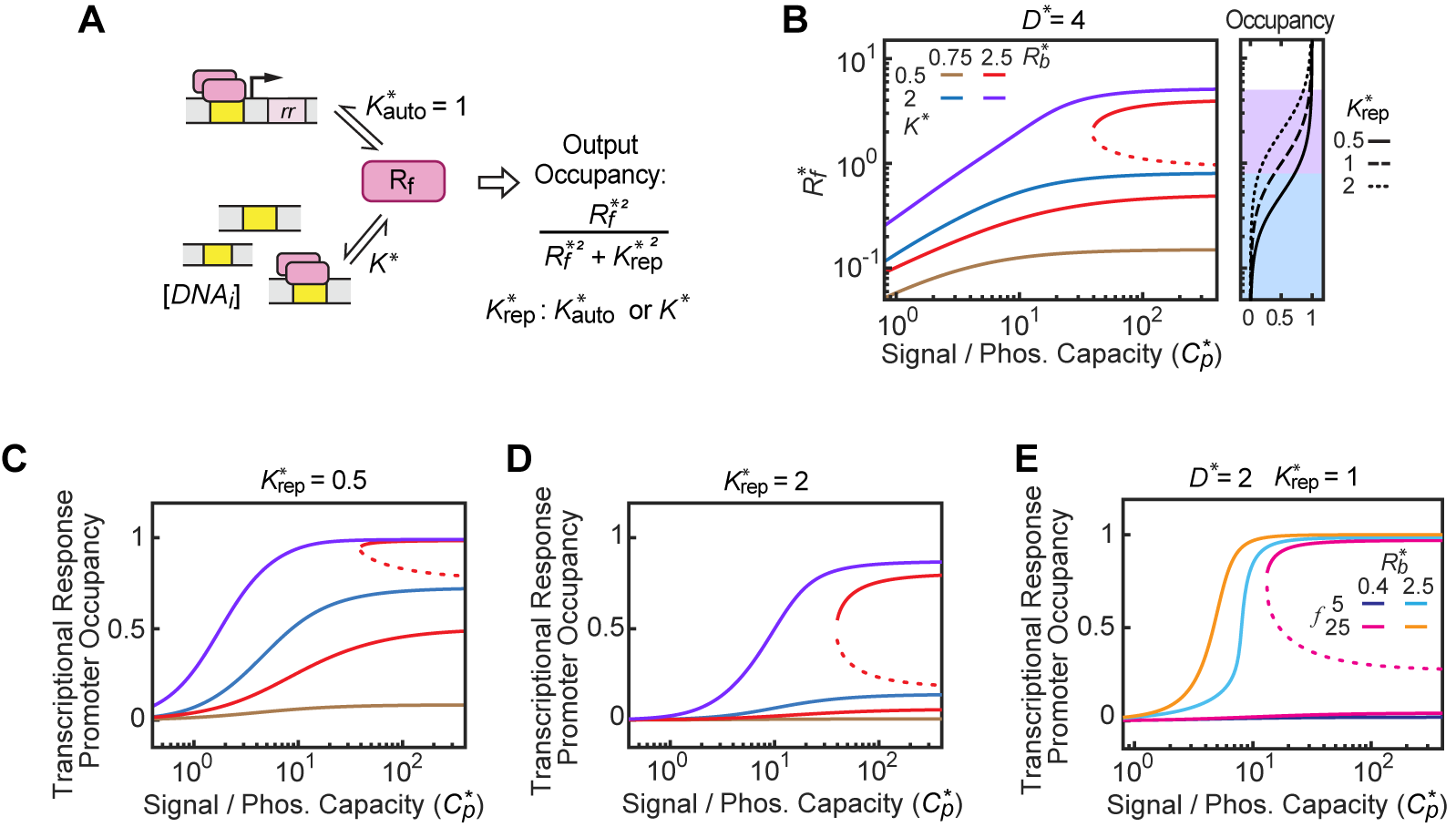

Supplement: S5 Fig — (A) Transcriptional response is defined as the promoter occupancy that is determined by Rf* and the binding affinity K*rep. K*rep will be different for different promoters, such as the autoregulated RR promoter and the RR-regulated promoters that contain sites among the competing TFBSs. (B-D) Impacts of Rb* and K* on Rf*, and subsequently the promoter occupancy. For systems with identical parameter sets (f = 5, D* = 4 and K* = 2), Rb* determines the concentration range of Rf* levels (blue or purple shaded areas in B) and the range of promoter occupancy in response to signals. In comparison to a weak TFBS affinity (K* = 2), a strong TFBS affinity (K* = 0.5) always leads to stronger TFBS competition, thus lower Rf* and lower promoter occupancy (C and D). For high Rb* and low K*rep values, such differences can be small, and both (purple and red lines in C) can reach near full occupancy. (E) Impacts of Rb* and f on transcriptional responses. High f can result in bistable responses (pink) while low f gives monostable but extremely weak responses (navy). (TIF) [file pcbi.1010738.s006.tif]

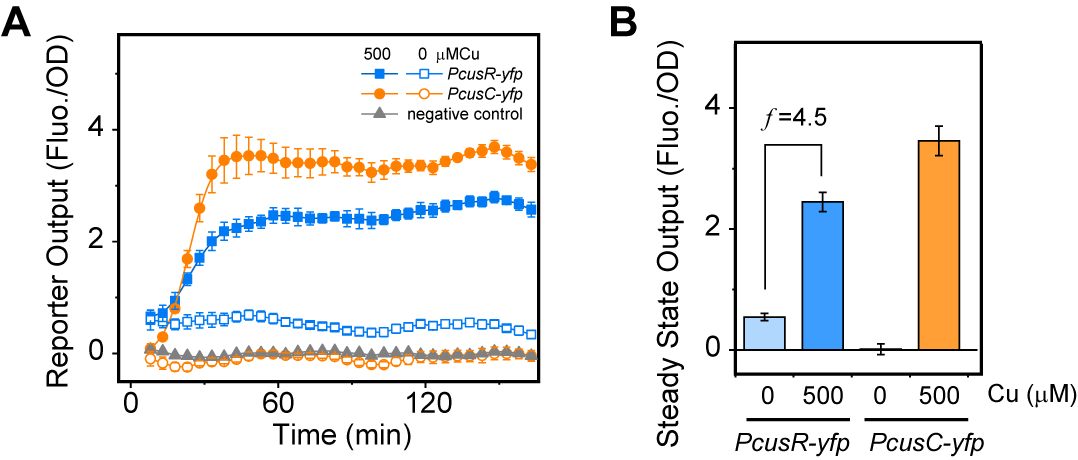

Supplement: S6 Fig — (A) Time course of YFP reporter output. BW25113 carrying pJZG209 (PcusR-yfp), pJZG157 (PcusC-yfp, positive control for Cu response) and pCL1920 (negative control with no yfp) were assayed for response to 0 or 500 μM CuSO4. Fluorescence normalized by OD600 was used as reporter output, with mean and standard deviations (std) from eight replicate wells shown. Results are from one representative of three independent experiments. (B) Estimation of the fold change. Steady-state output was computed as the average of output in the plateaued region (~60–120 min) of the time course data. Mean and std from three independent experiments are illustrated as bar graphs. (TIF) [file pcbi.1010738.s007.tif]

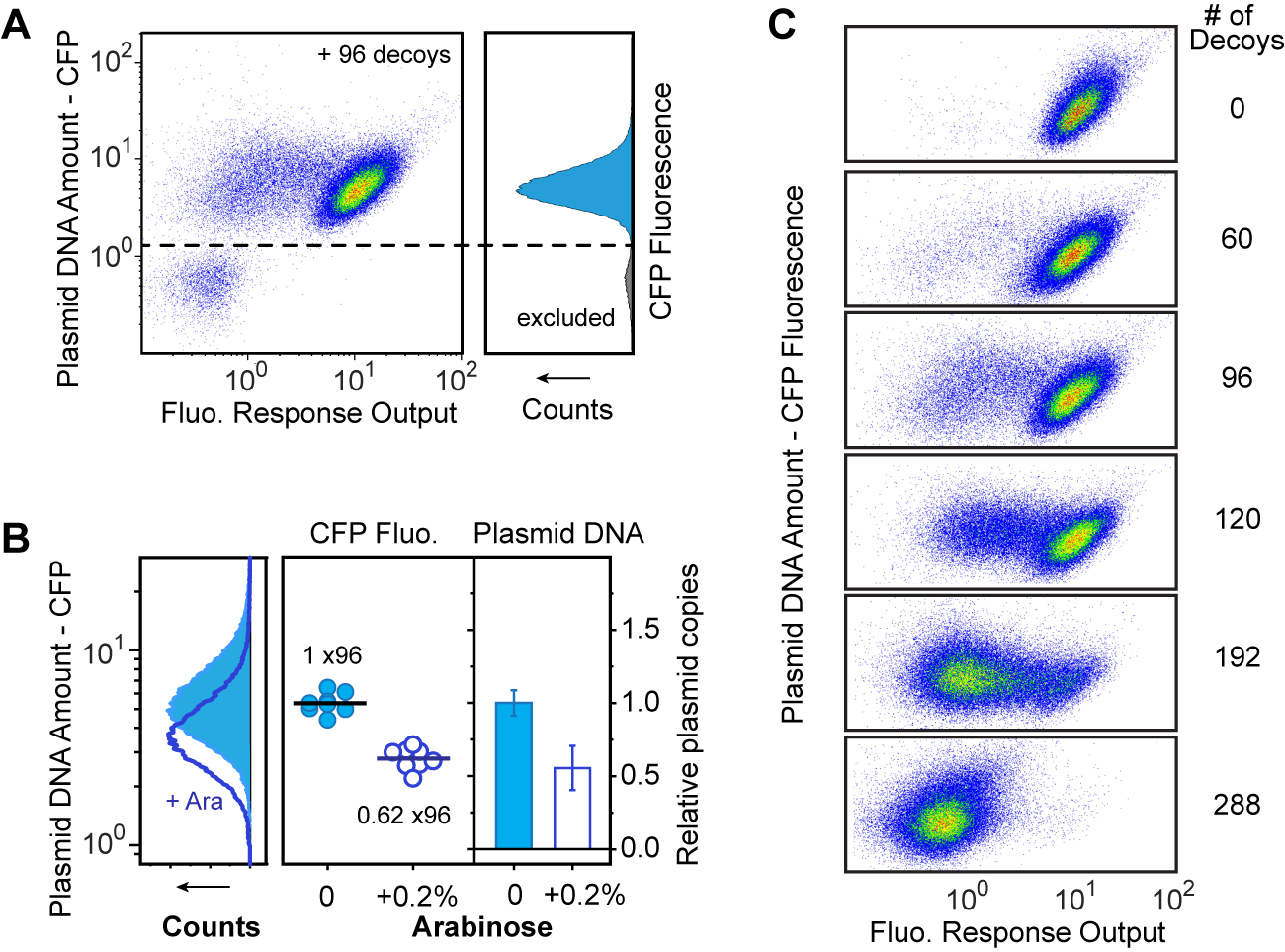

Supplement: S7 Fig — RU2118 (PcusC-mGreenLantern) carrying different decoy plasmids were assayed. All decoy plasmids carry a constitutively expressed CFP used for gating of the flow cytometry data (A) and estimation of plasmid copy number (B). A small population of cells with only background CFP fluorescence was often observed, especially in the presence of high concentrations of toxic CuSO4. None of these cells showed GreenLantern (GL) fluorescence. They are considered as dead cells or cells with impaired gene expression, thus were gated out with CFP histograms and excluded from further analyses. (B) Plasmid copy number estimation. The copy number of decoy plasmids has been determined as 96 in previous studies [18]. Addition of 0.2% arabinose (Ara) can inhibit replication of the plasmid origin and reduce the copy number, leading to reduced CFP fluorescence (left panel). The median of CFP fluorescence was used to derive the relative fluorescence. Solid and open circles represent CFP fluorescence of independent samples in the absence and presence of arabinose (middle panel). Arabinose reduced fluorescence to 62%, thus the copy number is estimated to be 0.62✕96≈60. Plasmid DNA extracted from corresponding cultures showed a similar value of the relative DNA amount (right panel), consistent with the relative copy number estimated from CFP. (C) Dot plots showing GL and CFP fluorescence of individual cells in response to 500 μM CuSO4. Decoy numbers are computed based on the plasmids and conditions used as follows: 0, pLH10 (0 sites); 60, pCusRBS1 (1 site, +Ara); 96, pCusRBS1; 120, pCusRBS2 (2 sites, +Ara); 192, pCusRBS2; 288, pCusRBS3. (TIF) [file pcbi.1010738.s008.tif]
