# Supplementary material for: Exploring the mono-/bistability range of positively autoregulated signaling systems in the presence of competing transcription factor binding sites
Source: PLoS Comput Biol. 2022 Nov 22;18(11):e1010738. doi: 10.1371/journal.pcbi.1010738 (PMC9725139; doi:10.1371/journal.pcbi.1010738)
Supplement: Attachment — Submitted filename: plosCB_Gaoetal_review.docx [file pcbi.1010738.s009.docx]

Summary: In this study, the authors dissect the bifurcation behavior of a TCS with an added module of transcription factor binding site competition. After a thorough consideration of each independent module (phosphorylation, competition, and auto-regulation), the authors present design rules for mono- and bistability in an auto-regulated TCS. They close the loop by experimentally validating their conclusions in an E. coli TCS by introducing different levels of decoy binding sites for the transcription factor and observing the distribution of a fluorescent reporter.

Overall, this manuscript presents a thorough analysis of an often-overlooked set of interactions, explores the conclusions experimentally, and adds to the bacterial TCS literature. I have a few major and minor comments.

187-189: In principle this could be any non-specific binding of the TF. This might be out of scope for this study, but non-housekeeping sigma factors for example (which have a fundamentally different activation/regulation processes, with potential for ultra-sensitivity in the module as opposed to the phosphorylation module for a TCS) also have multiple binding sites and can be auto regulated, can this analysis of TFBS competition promoting bistability in some parameter ranges be generalized to non-cooperative binding (h = 1) or without the phosphorylation module (which has LG <= 1 anyway)?

Eq S25: Nomenclature can get somewhat confusing. RRp in SI is Rf in main text? Rp = RRp-free i.e. Rf + DNA bound RRp? Is Rp or Rf the same as RRp in SI?

203-204: As Rp* >> K*, nearly all increase in Rp must go to Rf, therefore LG must come back to 1 (Figure 3C). Rp* = K* is clearly not the concentration where LG is maximal, so is there a specific value of Rp*/K* at which LG_B is maximal (for a given D* and hl), and any rationale for this value? In other words, at what fractional occupancy of a pool of TFBSs with a certain relative affinity (K*) to the auto regulated site does free phosphorylated response regulator gain the largest wrt total phosphorylated RR in this independent module?

220-222: Can the authors comment on how this bistability is different from the dead-end complex formation or TF sequestration bistability models described in ref 10?

222-224: In the limit of weak TFBS competition (K* >> 1) LG_B —> 1. In this range the phosphorylation + autoregulation module together still retain the ability to have a bistable response. Perhaps should be rephrased?

227-243: Selecting for high K* to ensure graded response in an auto activating circuit is an interesting take-away. Assuming that some of the TFBSs are promoter sites for downstream signaling in response to external cues for a TCS, there seems to be a trade-off in the information carrying capacity (I.e. resolution of disparate signals) of a channel (which would require a low K* perhaps?) and avoiding bistability (high K*). In other words, can low affinity for non-auto regulated binding sites for TCSs as a general selection criterion leave cells with more promiscuity, cross talk, or less specificity in signaling? Can the authors comment on such a trade-off? If two positively auto regulated TCSs both with a high K*, recognize different signals (Cu and Mg for example), how does this affect the information carrying capacity of the channel (<https://doi.org/10.1016/j.cels.2018.12.006>) as opposed to a low K* value? This analysis may add a layer of generality to this study.

Fig 4: Interestingly, PhoP in E coli seems to have a higher affinity for a non-auto regulated site (promoter site for mgrB through which a negative feedback is regulated, Miyashiro & Goulian, PNAS 2007 104(41) ), and it also has an estimated auto regulation strength of f ~ 20 (Ram & Goulian 2013 Plos genetics, Rao & Igoshin Plos CB 2021). Furthermore, removing the phosphatase capacity of PhoQ promotes bistability. Is it possible to study this TCS and include in Fig 4 as a potential counterexample? Potentially a conclusion would be that a negative feedback avoids bistability for this TCS.

PhoBR in E coli also has a weaker binding negative autoregulation site allowing for selection of stronger autoregulation (Gao & Stock, Cell Reports 2018). While that has dynamical benefits as the authors have shown, maybe PhoBR auto-repression may be revisited in light of the TFBS competition model in this study.

In summary, adding an example that bucks this trend shown in this figure and study, needing additional negative regulation to suppress bistability can improve the study.

308: Can the authors quantify the affinity of the decoy binding site relative to the autoregulated? Given the previous analysis of the role of K* and maxLG Rf* values aligning for bistability, an estimation of the CusRS system and the decoy sites might help the reader place the experiments in the phase plots in figures 3 or 6 for example.
