# Supplementary material for: Exploring the mono-/bistability range of positively autoregulated signaling systems in the presence of competing transcription factor binding sites
Source: PLoS Comput Biol. 2022 Nov 22;18(11):e1010738. doi: 10.1371/journal.pcbi.1010738 (PMC9725139; doi:10.1371/journal.pcbi.1010738)
Supplement: Attachment — Submitted filename: comments-1103.docx [file pcbi.1010738.s010.docx]

We thank the reviewers for these valuable comments and have made major revisions to the manuscript. Additional analyses of coupled negative feedback have been provided to study how mono-/bistability is affected by coupled negative feedback, which has been commonly observed for TCSs. We have also included additional modeling on the steady-state transcriptional responses. This allows us to discuss the trade-offs of maintaining monostability with specific parameters values. Detailed responses to the reviewers’ comments are provided below.

*Reviewer's Responses to Questions*

*Reviewer 1*

*Summary: In this study, the authors dissect the bifurcation behavior of a TCS with an added module of transcription factor binding site competition. After a thorough consideration of each independent module (phosphorylation, competition, and auto-regulation), the authors present design rules for mono- and bistability in an auto-regulated TCS. They close the loop by experimentally validating their conclusions in an E. coli TCS by introducing different levels of decoy binding sites for the transcription factor and observing the distribution of a fluorescent reporter.*

*Overall, this manuscript presents a thorough analysis of an often-overlooked set of interactions, explores the conclusions experimentally, and adds to the bacterial TCS literature. I have a few major and minor comments.*

*187-189: In principle this could be any non-specific binding of the TF. This might be out of scope for this study, but non-housekeeping sigma factors for example (which have a fundamentally different activation/regulation processes, with potential for ultra-sensitivity in the module as opposed to the phosphorylation module for a TCS) also have multiple binding sites and can be auto regulated, can this analysis of TFBS competition promoting bistability in some parameter ranges be generalized to non-cooperative binding (h = 1) or without the phosphorylation module (which has LG <= 1 anyway)?*

We agree with the reviewer that detailed discussions on this issue are beyond the scope for this study. We include a brief discussion in S1 Text (line S 83-87) to address this. We believe that the framework developed in this manuscript can be extended to other systems with non-cooperative binding. Once adjusted for binding stoichiometry, eq. S12 can be applied to non-cooperative binding, and LG*_B_* can be larger than 1 even with the binding cooperativity equal to 1.

*Eq S25: Nomenclature can get somewhat confusing. RRp in SI is Rf in main text? Rp = RRp-free i.e. Rf + DNA bound RRp? Is Rp or Rf the same as RRp in SI?*

[RRp] in S1 Text is *Rp* in the main text. We revised S1 Text to emphasize this (lines S 140-141). For simplicity, we assume that the HK has the same phosphatase activity toward free phosphorylated RR (*RRp-free*) and DNA-bound phosphorylated RR (*RRp-bound*), so the phosphorylation module can be decoupled from DNA binding and TFBS sequestration will not impact phosphorylation. This rationale has been provided in S1 Text.

*203-204: As Rp* >> K*, nearly all increase in Rp must go to Rf, therefore LG must come back to 1 (Figure 3C). Rp* = K* is clearly not the concentration where LG is maximal, so is there a specific value of Rp*/K* at which LG_B is maximal (for a given D* and hl), and any rationale for this value? In other words, at what fractional occupancy of a pool of TFBSs with a certain relative affinity (K*) to the auto regulated site does free phosphorylated response regulator gain the largest wrt total phosphorylated RR in this independent module?*

We provided additional analyses on the maximal LG*_B_* in S1 Text (lines S 88-97). A qualitative rationale has been included in the main text (line 206-207). The value of *K*/R_f_** at which LG*_B_* reaches the maximum is the root of a polynomial and can be numerically calculated. The corresponding occupancy of TFBSs for parameter values shown in Fig 3 has been computed, and it appears that LG*_B_* reaches the maximum when most of the TFBSs (~90%) are bound.

*220-222: Can the authors comment on how this bistability is different from the dead-end complex formation or TF sequestration bistability models described in ref 10?*

Comparison of the two bistability mechanisms have been added to the Discussion (lines 424-430).

*222-224: In the limit of weak TFBS competition (K* >> 1) LG_B —> 1. In this range the phosphorylation + autoregulation module together still retain the ability to have a bistable response. Perhaps should be rephrased?*

The sentence has been rephrased to “the system can lessen the bistability-promoting effect of TF sequestration with a high *K^*^* value” (lines 226-227).

*227-243: Selecting for high K* to ensure graded response in an auto activating circuit is an interesting take-away. Assuming that some of the TFBSs are promoter sites for downstream signaling in response to external cues for a TCS, there seems to be a trade-off in the information carrying capacity (I.e. resolution of disparate signals) of a channel (which would require a low K* perhaps?) and avoiding bistability (high K*). In other words, can low affinity for non-auto regulated binding sites for TCSs as a general selection criterion leave cells with more promiscuity, cross talk, or less specificity in signaling? Can the authors comment on such a trade-off? If two positively auto regulated TCSs both with a high K*, recognize different signals (Cu and Mg for example), how does this affect the information carrying capacity of the channel (*[*https://doi.org/10.1016/j.cels.2018.12.006*](https://doi.org/10.1016/j.cels.2018.12.006)*) as opposed to a low K* value? This analysis may add a layer of generality to this study.*

We thank the reviewer for this comment. The signaling capacity will depend on steady-state responses. One section and two figures (Fig 7 and S5) have been added to discuss the steady-state transcriptional response affected by different parameters (lines 324-350). Analyzing transcriptional responses allows us to discuss the potential costs or trade-offs for maintaining monostability with specific parameter values (lines 459-469).

Specific to the binding competition, the values of *K** will have an impact on the levels of transcriptional responses, which ultimately determine the signaling capacity. Transcriptional responses of the autoregulated system will depend on both *K** and the steady-state level of *R_f_**. Higher *K** can potentially result in lower promoter occupancy based on the binding equilibrium, but it can also lead to less TFBS competition, thus higher steady-state *R_f_** levels to increase the response. So, *K** will have complicated effects on the responses and signaling capacity. Effects of *K** on transcriptional responses will depend on other parameters, particularly the protein production rate of the TF. We added a section in S1 text (lines S 172-189) to address this.

We believe that the binding affinity for different TF-regulated genes are likely to be optimized differently for expression levels and temporal dynamics for individual genes. A universal low affinity is unlikely to be a general selection criterion. However, selecting a strong affinity for the autoregulated promoter can also effectively increase the values of *K** and promote monostability. And this is what has been observed based on the current global binding studies (Fig 4). Thus, we focus on the binding affinity of the autoregulated promoter. We also included discussion on how binding competition reduces the signaling capacity (Lines 340-341, S1 Text S 183-189). We think that analyzing the information capacity of duplicated signaling systems is beyond the scope of this study.

*Fig 4: Interestingly, PhoP in E coli seems to have a higher affinity for a non-auto regulated site (promoter site for mgrB through which a negative feedback is regulated, Miyashiro & Goulian, PNAS 2007 104(41) ), and it also has an estimated auto regulation strength of f ~ 20 (Ram & Goulian 2013 Plos genetics, Rao & Igoshin Plos CB 2021). Furthermore, removing the phosphatase capacity of PhoQ promotes bistability. Is it possible to study this TCS and include in Fig 4 as a potential counterexample? Potentially a conclusion would be that a negative feedback avoids bistability for this TCS.*

*PhoBR in E coli also has a weaker binding negative autoregulation site allowing for selection of stronger autoregulation (Gao & Stock, Cell Reports 2018). While that has dynamical benefits as the authors have shown, maybe PhoBR auto-repression may be revisited in light of the TFBS competition model in this study.*

*In summary, adding an example that bucks this trend shown in this figure and study, needing additional negative regulation to suppress bistability can improve the study.*

We thank the reviewer for this suggestion. Additional modeling with a coupled negative feedback has been added (Fig 6CD, S4 Fig and lines 309-322). Indeed, a coupled negative feedback always reduces LG and suppresses bistability. We included modeling of the simple scheme, in which the TF binds to both activation and repression sites within its own promoter, and discussed a more complex scheme, such as occurs with PhoP. References for these examples as well as the more comprehensive analyses of all modes of coupled feedback (Tiwari and Igoshin 2012) have been cited.

As for the fact that PhoP in *E. coli* has a higher affinity to *mgrB* than the autoregulated *phoP* promoter, we do not consider it necessarily as a counterexample. As shown in Fig4A, for PhoB, PhoP (Salmonella) and BvgA, there are genes with a higher information content/affinity (grey circles) than the autoregulated promoter (pink diamond). As long as the affinity to the autoregulated promoter is among the highest of all TFBSs, it can potentially suppress the bistability caused by TF competition and sequestration. Information content analyses of TFBSs require a well-curated and accurate database for all TFBSs or global binding studies with high resolution, e.g., ChIP-exo. We are not aware that such data are readily available for *E. coli* PhoP.

*308: Can the authors quantify the affinity of the decoy binding site relative to the autoregulated? Given the previous analysis of the role of K* and maxLG Rf* values aligning for bistability, an estimation of the CusRS system and the decoy sites might help the reader place the experiments in the phase plots in figures 3 or 6 for example.*

Quantifying the affinity of CusR to DNA will certainly help evaluation of the CusRS response, but we think it will not significantly alter the conclusions of this study. Biochemical characterization of the binding of CusR to DNA will not be trivial, especially given that there are conflicting reports on the location, geometry and cooperativity of CusR binding sites. Differences between the decoy and the CusR binding site in its promoter, and its potential impact have been explained in S1 Text (lines 214-221).

*Reviewer #2: The author studied the autoregulatory two component systems combined with modular models and derivation of relationships between system’s mono-/bi-stability with transcription factor binding sites (TFBSs) competition, phosphorylation capacity, and fold changes. In particular the TFBSs competition and phosphorylation activity can shift the system from monostability towards space that is bistability prone. The models is well constructed, the derivation is quite elegant, and the experiments is straightforward and supporting the discoveries from the theory. Honestly, I quite enjoy reading the paper. I would recommend to publish this manuscript. There are some really minor points, they are not necessarily required to addressed in this manuscript (except the first one) but I would like to know how the authors would respond. I put them in the following:
1. Figure 1A, the middle box, it is a bit misleading with Pink box as Rf then the output of the middle module as Rf as well.*

We thank the reviewers for raising this issue. Figure 1A and 3A have been modified to reflect *R_f_* as the output of the DNA binding module.

*2. In general, many signaling or regulatory systems evolves towards bistability, ultrasensitive and bistable systems can usually serve as a noise filter to increase the fidelity of responding to fluctuating environments. Also, it should be much easier for a system to achieve monostability because instability requires both particular structures in corresponding parameter regime. But it seems that the authors think the systems are avoiding to become bistability, why uniform graded responses are needed from an evolutionary point of view?*

We agree that some signaling systems have evolved toward ultrasensitivity and bistability, but these features are not always advantageous for many signaling systems. A graded response in TF activation can establish a continuity of gene expression levels in response to different signal strengths. This can certainly be beneficial because cells may not need to use all the resources to elicit the full response when the signal is weak. Moreover, a graded response may also allow a system to turn on different sets of genes dependent on signal strengths. It has been shown that many two-component systems display graded or continuous responses despite of the positive autoregulation, which is often associated with bistability. Our study tries to explore the parameter regime that allows positively autoregulated TCSs to achieve monostable and graded responses.

We modified paragraph 2 in the introduction (lines 88-94) to emphasize above points.

*3. Another interesting questions is that what if the other TFBSs are actually functional in other TCSs, (i.e. there are cross-talks between different TCSs) will that affect the rule discovered here for the overall responses?*

If some TFBSs are promiscuous and can be bound by other TCS TFs or other DNA-binding proteins, we believe this will change the DNA binding competition and thus, the TF sequestering effects. However, such impact will be significant only if the two systems involved in cross-talk are sharing a large number of promiscuous binding sites and are simultaneously activated for DNA binding. We consider such conditions to be fairly uncommon and likely to occur only for some global regulators or nucleoid-associated proteins. We modified the text in the Discussion (lines 476-479) to acknowledge these effects.

*Reviewer #3: This manuscript contains a (mostly theoretical) study of positively autoregulating two-component signaling systems. Earlier work has studied such systems extensively; this work, however, extends these analyses by considering competition between the transcription factor binding sites responsible for the autoregulation and other binding sites or "decoys". The presence of decoys can enhance the sensitivity of the feedback, and hence can result in bistable states under parameter regimes where the state is monostable in the absence of decoys. The goal of the manuscript is to determine the conditions for mono- and bistability in the presence of decoys. A limited experimental test of the theory is presented in which decoys are added to an existing system and its effect on the response is qualitatively compared to theory.

Technically, I think the study is well conducted and the manuscript is for the most part clearly written. Nevertheless I do have a few suggestions for improvement.

1. The authors have chosen an approach based on deterministic equations, even though it has been shown that stochasticity can affect bistability and bimodality and some previous models have used a stochastic approach to describe the effect of decoys (Burger et al). The authors should at least discuss the limitations of that approach. Also, I could not find one of the relevant references in this regard:

Anat Burger, Aleksandra M. Walczak, and Peter G. Wolynes
Influence of decoys on the noise and dynamics of gene expression
Phys. Rev. E **86**, 041920.*

We acknowledged the limitation of the deterministic approach in the last paragraph of the Discussion and added the reference as suggested (lines 472-476). At the start of Methods (lines 488-490), we also added an explanation that choosing the deterministic approach can simplify the modeling of the complex reaction networks of phosphorylation/dephosphorylation of TCSs.

*2. The function of two-component systems is (presumably) to allow cells to respond to changes in an environmental signal. Hence, I would say the "response" of the system is a function that describes the steady-state value of $R_T$ (or perhaps $R_P$) as a function of that signal. The authors seem to use the term "response" differently, because they sometimes state that the system has a mono- or bistable response where I would say they only showed that it is mono- / bistable for the given parameters and signal level. I recommend avoiding the term response in those cases.*

We thank the reviewer for raising this issue. We have modified the term “response” throughout the manuscript to minimize the confusion. In the revised manuscript, the term "response” specifically refers to the steady-state transcriptional output of the signaling system, which is modeled as TF occupancy of the promoter of interest and ultimately determined by Rf and the TF affinity (lines 336-337). And one section has been added to discuss impacts of different parameters on transcriptional responses (lines 324-350).

*3. Very much related to the previous point: throughout the manuscript I was sometimes confused because the analysis seems to (correctly) identify parameters/conditions that allow a monostable state, without verifying whether (a) the parameters would still allow the system to (significantly) respond to the environmental signal, and (b) bistability was avoided along the complete response curve (e.g., for all Cu2+ concentrations). For example, it is concluded that bistability can be avoided by choosing a small fold change $f$; but a small $f$ may also imply a weak response to changes in the signal. A similar point can perhaps be made about the parameter $Cp$. What I mean is: can the authors explain how the signal affects the system and how the parameters affect the over-all response to the signal? This may also help in connecting to the experiments, where the signal is the independent variable that has been manipulated.*

See comments above about the definition of the term “response”. We agree with the reviewer that the bistability range shown is only the parameter space that allows bistable output. The actual transcriptional output will depend on other parameters, such as *R_b_* or TF affinity to the promoter of interest. *R_b_* has an important role in setting the range of TF concentrations *R_T_* and *R_f_*. We agree that a small fold change *f* coupled to a low RR production rate *R_b_* can result in an extremely weak response to signals, which may represent the cost of avoiding bistability. The overall response will depend on individual parameters.

We added two figures (Fig 7 and S5) to model the transcriptional output affected by different parameters and show how signal changes the response, i.e., promoter occupancy. One section has been devoted to discussion of the transcriptional response (lines 324-350). Signals are known to modulate the phosphorylation and dephosphorylation reactions rates, thus are modeled as a change of *Cp** (lines 257-258, 281-284). Dependence of steady-state responses on *Cp** have been used to show the complete response curve.

*\## Minor comments
4. In Eq. (1) (and the corresponding equation in the supplement) the parameter $k_{dil}$ is eliminated after the second equality sign. Obviously, the left-hand and right-hand side of this equality sign are not equal; this is incorrect. (After the differential equation is equated to zero to find the equilibria, the $k_{dil}$ *can* be divided out, so this error has no consequences, but it should nevertheless be fixed.)*

Eq.(1) has been modified as suggested.

*5. In line 145, I think "the necessary condition" should be "a necessary condition"; I'm sure we can state many other necessary conditions.*

We agree with the reviewer and have changed “the” to “a” (line 146).

*6. In lines 154-156 the reader gets the impression that the condition that LG3 > 1 is not only a necessary condition but also a sufficient one. This, I believe, is misleading: there are further requirements on $R_b^*$ , $K_{auto}$, and other parameters.*

The text has been modified to emphasize that “an LG*_3_* value larger than 1 is a necessary condition for bistability” (line 157).

*\## Very minor comments

The comments below purely cosmetic (but easy to fix).

7 In several sentences, articles (the, a/an) seem to be missing.*

Corrected throughout the manuscript.

*8 In the equations, sometimes notation could be improved.

* The symbol * is not the mathematical symbol for multiplication.*

Corrected, the symbol * for multiplication has been removed.

** In equation (2) I see no reason to use partial derivatives.*

Corrected, derivatives rather than partial derivatives are used.

** Variables consisting of multiple characters, such as $LG$ for linear gain, $HK$ for histidine kinase concentration, and so on, read as multiplications ($H \cdot K$). In fact, the software used to typeset the equations interprets them as such and therefore inserts spaces between these symbols. It is in my opinion better replace them by a single symbol (possibly with a subscript label.*

Some of the variables with multiple letters, such as ‘*hl*”, have been changed to single letter variables, e.g. ‘*l*’. We understand that variables with multiple characters may cause confusion, but we believe they are easier to associate with the biochemical species or characteristics of the particular variable. To reduce potential confusion, we modified all the equations using non-italicized fonts (e.g., HK or RR) to represent individual species and “[]” to denote the concentration variables. We also changed italicized *LG* to LG in Roman to differentiate from single letter variables.

** Italic characters are reserved for variables or parameters, and hence standard functions such as $\log(x)$ are normally set in Roman. Subscript labels should be Roman to distinguish them from indices (which are variables that can take on multiple values).*

We thank the reviewer for this suggestion. ‘log’, subscripts in *K*_auto_, *K*_dil_ and other relevant labels have been corrected.
